# Medicinal Plants Qua Glucagon-Like Peptide-1 Secretagogue via Intestinal Nutrient Sensors

**DOI:** 10.1155/2015/171742

**Published:** 2015-12-15

**Authors:** Ki-Suk Kim, Hyeung-Jin Jang

**Affiliations:** College of Korean Medicine, Institute of Korean Medicine, Kyung Hee University, 1 Hoegi-dong, Dongdaemun-gu, Seoul 130-701, Republic of Korea

## Abstract

Glucagon-like peptide-1 (GLP-1) participates in glucose homeostasis and feeding behavior. Because GLP-1 is rapidly inactivated by the enzymatic cleavage of dipeptidyl peptidase-4 (DPP4) long-acting GLP-1 analogues, for example, exenatide and DPP4 inhibitors, for example, liraglutide, have been developed as therapeutics for type 2 diabetes mellitus (T2DM). However, the inefficient clinical performance and the incidence of side effects reported on the existing therapeutics for T2DM have led to the development of a novel therapeutic strategy to stimulate endogenous GLP-1 secretion from enteroendocrine L cells. Since the GLP-1 secretion of enteroendocrine L cells depends on the luminal nutrient constituents, the intestinal nutrient sensors involved in GLP-1 secretion have been investigated. In particular, nutrient sensors for tastants, cannabinoids, and bile acids are able to recognize the nonnutritional chemical compounds, which are abundant in medicinal plants. These GLP-1 secretagogues derived from medicinal plants are easy to find in our surroundings, and their effectiveness has been demonstrated through traditional remedies. The finding of GLP-1 secretagogues is directly linked to understanding of the role of intestinal nutrient sensors and their recognizable nutrients. Concurrently, this study demonstrates the possibility of developing novel therapeutics for metabolic disorders such as T2DM and obesity using nutrients that are readily accessible in our surroundings.

## 1. Treatment of Type 2 Diabetes Mellitus

Diabetes is a metabolic disease characterized by high blood glucose levels caused by insufficient insulin production or insulin resistance. While type 1 diabetes mellitus (T1DM) is considered an autoimmune disease caused by pancreatic *β* cell destruction, type 2 diabetes mellitus (T2DM) is caused by lifestyle factors, such as age and obesity, and by insulin resistance, where the body cells fail to respond to insulin, and accounts for 90–95% of all diabetes cases [1].

Accumulating evidence implicates that chronic hyperglycemia caused by diabetes results in tissue damage and furthermore increases the risk of micro- and macrovascular diseases, sclerosis of the arteries, cardiovascular disease, diabetic kidney disease, and retinal disease [2]. According to the “Diabetes Fact Sheet in Korea 2013” published by the Korean Diabetes Association, 44.4% of diabetes patients have obesity, 54.6% have hypertension, and 80% have dyslipidemia.

Various therapeutic strategies for T2DM had been developed including stimulating insulin secretion (sulfonylurea), decreasing glucose release from the liver (biguanides), reducing carbohydrate absorption in the gastrointestinal (GI) tract (*α*-glycosidase inhibitor), and enhancing the susceptibility of insulin receptor (thiazolidinediones) [3, 4]. However, side effects and gradually declining efficacy are limitations of these strategies (Table 1).

T2DM therapeutics that regulates blood glucose and weight gain while restoring and enhancing *β* cell function is a potentially ideal strategy. In the last decade, incretin-based therapy has been spotlighted as a substitute strategy to circumvent the limitations of existing therapies.

## 2. GLP-1 Secretagogue for Type 2 Diabetes Treatment

Incretin is a gut hormone secreted from the small intestine during a meal and stimulates insulin secretion from the pancreatic *β* cells. Glucagon-like peptide-1 (GLP-1) is a typical incretin hormone and is produced by differential posttranslational processing of proglucagon in the gut and brain [5]. GLP-1 secreted from enteroendocrine L cells plays various physiological roles in glucose homeostasis and feeding behavior. GLP-1 slows gastric emptying, inhibits gut motility, and suppresses appetite while it increases *β* cells proliferation, enhances *β* cells function, and stimulates pancreatic insulin secretion [5, 6]. The insulin stimulating effect of GLP-1 is tightly regulated by the blood glucose concentration. The insulin stimulating effect is abrogated when the blood glucose concentration is under 4.5 mmol/L and the half maximal effective concentration of GLP-1 is about 10 pmol/L [7]. Therefore, GLP-1 receptor agonists minimize the risk of hypoglycemia, reduce food intake, and lead to weight loss [8]. Indeed, lowered plasma active GLP-1 levels observed in T2DM patients support the GLP-1 receptor agonism as an appropriate therapeutic strategy [9]. Direct administration of GLP-1 restored initial insulin dyscrinism and showed a rapid blood glucose lowering effect [9]. However, direct administration of active GLP-1 has not been applied in clinical medicine because of its short half-life (<2 min) due to the enzymatic cleavage of dipeptidyl peptidase-4 (DPP4).

On the basis of the efforts to improve the GLP-1 activity (both the benefits and the limitations), incretin-based therapies, such as DPP4 inhibitors and stable GLP-1 analogs, have been developed in recent years. These incretin-based therapies show fewer side effects related to hypoglycemia and weight gain than existing non-incretin-based medications and even regenerate pancreatic *β* cells [8]. Nevertheless, inadequate effectiveness and causing pancreatitis are reported to be the limitations of the incretin-based therapies [10] (Table 1). GLP-1 analogues, such as exenatide and liraglutide, have similar effects to GLP-1 due to their binding to the GLP-1 receptor with greatly extended durability [11, 12]. However, patients prescribed GLP-1 analogues have suffered needle phobia and side effects such as nausea, vomiting, and anorexia [13, 14]. DPP4 is a ubiquitous enzyme involved in the enzymatic cleavage of more than 20 different endogenous peptides in humans [15]. Thus, unclear side effects due to inhibiting endogenous peptides are a potential risk of DPP4 inhibitor prescription.

A possible strategy to improve the limitations of existing therapeutics for T2DM is directly stimulating GLP-1 secreting cells. This strategy is based on the activation of cell surface receptors and their cellular signal transduction pathway involved in GLP-1 secretion. These receptors for the GLP-1 secretagogues are found on the surface of enteroendocrine L cells and are able to respond to the luminal nutrient composition.

## 3. Nutrient Sensing in the Gut

**Figure 1:**
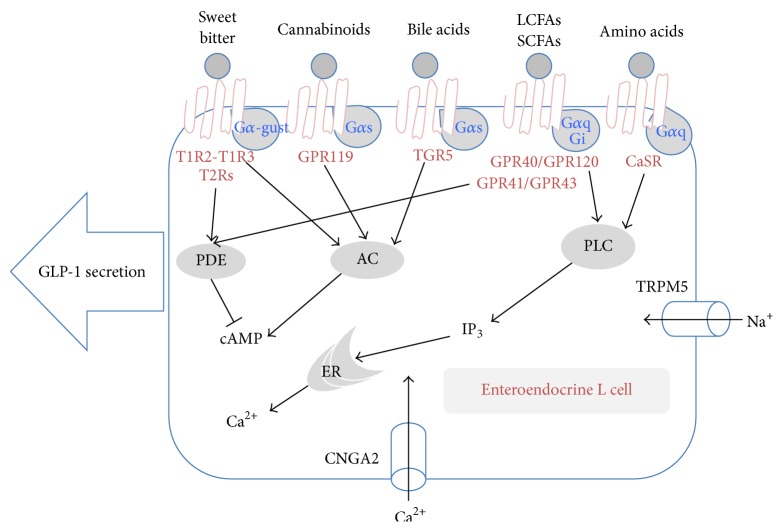
Nutrient sensors in the enteroendocrine L cells. Nutrient sensors existing in the endocrine cells and their possible signal transduction pathways. # LCFAs, long-chain fatty acids; SCFAs, short-chain fatty acids; PDE, phosphodiesterase; AC, adenylyl cyclase; PLC, phospholipase C; IP_3_, inositol 1,4,5-triphosphate; ER, endoplasmic reticulum.

The GI tract is a significant sensor for the ingested nutrients throughout its length [33]. Ingested foodstuffs are digested into small nutritional compounds and then absorbed by the cells located in the surface of the gut lumen. Distinguishing the nutrients is critical for determining the nutritive and toxic qualities of ingested material [34, 35]. In particular, enteroendocrine cells which secrete gut-peptides, such as GLP-1, glucose-dependent insulinotropic peptide (GIP), peptide YY (PYY), and cholecystokinin (CCK), respond to the luminal nutrients, such as tastants, fatty acids, amino acids, cannabinoids, and bile acids (Figure 1) [36–38]. In this review, nutrient sensors expressed in enteroendocrine L cells, GLP-1 secreting cells existing throughout the intestine, are discussed.

### 3.1. Sweet Taste Receptors

Natural sugars, such as mono-, di-, and oligosaccharides, are fundamental energy sources to most organisms. The sweet taste perception that occurs in the oral cavity is able to recognize the natural sugars and facilitates their ingestion into the GI tract. The sugar sensing event in the tongue is known to be due to a heterodimer of T1R2 and T1R3, which are the type 1 taste receptors [39]. Consequent activation of a taste specific G protein, gustducin, transmits the signal cascade through the transduction elements, such as phospholipase C (PLC), inositol 1,4,5-triphosphate (IP_3_), diacylglycerol (DAG), adenylyl cyclase (AC), and the Ca^2+^-sensitive transient receptor potential channel M5 (TRPM5) [34, 40]. In particular, the increase of intracellular second messengers, Ca^2+^ and cAMP, due to activation of adenylyl cyclase and IP_3_, respectively, is involved in the sweet taste perception [41, 42].

In enteroendocrine L cells, sweet taste receptor-mediated GLP-1 secretion appears to be due to activation of G*α*-gustducin. In human enteroendocrine NCI-H716 cells, glucose and sucrose only activate G*α*-gustducin while they inactivate G*α*i_1,2_ and/or do not affect G*α*s [43]. Moreover, abolished increase in the plasma GLP-1 and plasma insulin levels after oral glucose administration to G*α*-gustducin null mice supports the involvement of G*α*-gustducin in glucose sensing [43].

G*α*-gustducin is a G*α*-transducin-like G protein *α*-subunit expressed in 25~30% of taste receptor cells and is expected to activate PDE to decrease cAMP [41, 44]. Only 15% of G*α*-gustducin-positive cells are coexpressed with GLP-1 in the mouse jejunum [33]. Exact understanding of the role of G*α*-gustducin is challenging because of the following issues: (1) the low coexpression ratio of G*α*-gustducin with taste cells and GLP-1-positive cells; (2) frequently found coexpression of G*α*-gustducin with the other G protein *α*-subunits, such as G*α*i_1,2_, G*α*_15_, G*α*_16_, G*α*_q/11_, G*α*s, and G*α*-transducin; and (3) the ability of G*α*-gustducin to activate different molecules, which have the opposite function on the intracellular cAMP levels [34]. One possible hypothesis is that G*α*_15_ (mouse) and G*α*_16_ (human), which are able to couple with promiscuous GPCR, participate in the sweet taste perception [45].

Sweet taste receptors are expressed in the enteroendocrine L cells with its signal transduction elements, which are also found in the lingual sweet taste receptors [43]. The GLP-1 secretion stimulated by artificial sweeteners supports the existence of sweet taste perception in the enteroendocrine L cells.* In vitro* studies using human and mouse enteroendocrine cells demonstrated that sucralose stimulated GLP-1 and GIP secretion [43, 46]. However,* in vivo* studies using healthy human subjects demonstrated that intragastric infusion of artificial sweeteners, for example, sucralose, aspartame, or acesulfame K, or fructose does not stimulate GLP-1, PYY, or GIP release while only glucose stimulated GLP-1 and PYY release [47, 48]. Furthermore, a human study using a sweet taste receptor inhibitor demonstrated that the glucose has a GLP-1 and PYY secreting effect via the sweet taste receptor while blocking the sweet taste receptor did not affect the GLP-1 secreting effect of the liquid meal [49].

These results showing secretion of GLP-1 and PYY depends on the structural analogy to glucose lead us to deduce that medicinal plants, which abundantly contain glycosidic compounds, have a possibility of stimulating GLP-1 secretion through the activation of sweet taste receptors in the enteroendocrine L cells.

Ginsenosides, triterpenoid saponins that are found abundantly in* Panax ginseng*, have been classified into dammarane type and oleanane type according to their carbon skeletons of aglycone. Several ginsenosides, such as Rb1, Re, Rb2, C-K, and Rg3, were reported to have antidiabetic and antiobesity effects [22, 24, 25, 32, 50].

Long-term intraperitoneal injection of Rb1 (10 mg/kg) showed drastically decreased food intake, body weight, and body fat mass and also attenuated basal hyperglycemia in obese rats [22]. In this study, Rb1 increased c-Fos expression, a marker of neuronal activity, in the cells of nucleus of the solitary tract (NTS), ventromedial hypothalamic nucleus (VMN), and arcuate nucleus of the hypothalamus (ARC), all of which are brain parts known to be involved in the feeding behavior [22].

Intraperitoneal injection of Re (20 mg/kg) for 12 days reduced fasting blood glucose levels to 180 ± 10.8 mg/dL compared to the saline treated group (235 ± 13.4 mg/dL) in ob/ob mice [24].

Oral treatment of Rg3 (25 mg/kg) showed 9% of blood glucose reduction efficacy compared to the control mice group during the OGTT [25]. This glucose lowering effect of Rg3 seems to be due to its insulin secreting effect on the pancreatic *β* cell.

The ginsenosides Rb1, Rb2, Re, and Rg3 have glucose residues attached on the four-carbon ring structure dammarane backbone and thus have a possibility of stimulating GLP-1 secretion via the sweet taste receptor signal transduction pathway in the enteroendocrine L cells.

T1R3 and gustducin also contribute to glucose absorption from the enterocytes and affect the GLP-1 secreting ability of the enteroendocrine cells through the regulation of Na^+^-dependent glucose cotransporter 1 (SGLT1) expression [46]. Therefore, the role of sweet taste receptors in glucose homeostasis should be considered with the role of the enterocytes participating in glucose absorption.

### 3.2. Bitter Taste Receptors

Bitter tastants have not been considered as a fundamental nutrient but are abundantly found in medicinal plants, vegetables, alcohol beverages, and coffee. The bitter tastants can be recognized by the type 2 taste receptors (T2Rs) expressed in the tongue and small intestine. These bitter taste receptors are GPCR coupled with taste specific G protein gustducin identical to the sweet taste receptors [34].

The downstream signal transduction of the bitter perception triggered by the activation of gustducin closely resembles the sweet taste perception in the gut [51]. However, binding of the bitter tastant to its receptor results in a decrease of intracellular cAMP levels due to the activation of phosphodiesterase (PDE). In the bitter perception, the activation of PDE is due to the activation of G*α*-gustducin rather than G*α*i_1,2_ [38]. It is still controversial as to why G*α*-gustducin activates different intracellular molecules, AC and PDE, by the sweet and bitter stimuli, respectively. Moreover, the roles of both increased and decreased intracellular cAMP levels by each tastant binding have not been elucidated.

There are 25 deorphanized T2Rs expressed in human [52, 53]. These 25 T2Rs are also expressed in nonchemosensory cells, such as human airway smooth muscle cells and human enteroendocrine NCI-H716 cells, as well as the human tongue [38, 54, 55]. But the order of the mRNA expression levels of 25 T2Rs between these cells is quite different. This indicates that the bitter tastant recognized in the tongue might not be recognized as a bitter tastant in the gut. Indeed, denatonium benzoate, which is known to be the most bitter tastant in humans, shows a smaller GLP-1 secreting effect than quinine, another bitter tastant in human enteroendocrine NCI-H716 cells [38]. The different expression orders of T2Rs between the tongue and enteroendocrine cells may enable the bitter tastants to be novel GLP-1 secretagogues without inducing nausea and a repellent sensation from the tongue.

Quinine is a crystalline alkaloid that naturally occurs in the bark of the cinchona tree. It has been used as an antimalarial drug and a bitter flavor component of tonic water [56, 57].

Quinine exerts a GLP-1 secreting effect on human enteroendocrine NCI-H716 cells as well as the bitter taste receptor agonist denatonium [38]. A transcriptomic study demonstrated that several bitter taste receptors and GPCR pathway elements including PDE and IP_3_ receptor were upregulated in response to quinine treatment in the human enteroendocrine NCI-H716 cells [26].

In Korean traditional medicine, a disease known as “Sogal” shares the same pathological physiology with diabetes mellitus. According to the “Donguibogam: Principles and Practice of Eastern Medicine,” registered in UNESCO Memory of the World, bitter tasting medicinal plants are effective for the treatment of “Sogal.”

*Gentiana scabra* fulfills the categories of bitter tasting medicinal plants in the “Donguibogam” and, thus,* Gentiana scabra* extract is frequently prescribed for treatment of “Sogal” in Korean traditional medicine. Indeed,* Gentiana scabra* root extract exerts a GLP-1 secreting effect on the human enteroendocrine cells through the G protein *βγ*-subunit-mediated pathway, and as a result GLP-1 and insulin are released and attenuate hyperglycemia in a type 2 diabetic mouse model [30]. Another study demonstrated that an ethyl acetate fraction of* Gentiana scabra* root extract exerts the strongest GLP-1 secreting effects among evaluated fractions and upregulated the PDE, PLC, and IP_3_ receptors and DAG mRNA expression [58]. A mass spectrometry analysis revealed several bitter iridoid compounds contained in the* Gentiana scabra* extract and, among them, loganic acid exerts a GLP-1 secreting effect on the human enteroendocrine NCI-H716 cells [30].* Gentiana scabra* extract (100 mg/kg) decreased glucose levels of db/db mice during the OGTT. Plasma GLP-1 and plasma insulin levels also drastically increased 10 min after the oral administration of* Gentiana scabra* extract along with the glucose gavage [30].

*Anemarrhena asphodeloides*,* Bupleurum falcatum*, and* Citrus aurantium* also fulfill the categories of bitter tasting medicinal plants and have been reported to stimulate GLP-1 secretion [27–29]. In particular, a hexane fraction of the* Bupleurum falcatum* extract (100 mg/kg) significantly decreased blood glucose levels of db/db mice during the OGTT [28].

However, it is not clear whether these medicinal plant extracts activate the bitter taste receptor, due to a lack of functional studies on receptor activation.

Since quinine is used as a bitter flavor in tonic water and* Gentiana scabra* is categorized as a bitter herbal medicine, they clearly activate bitter taste receptors. Moreover, the fact that most medicinal plants have a bitter taste implies that the bitter taste receptor agonists are abundantly present in medicinal plants. However, as described above, not every bitter taste recognized at the tongue is also bitter to the gut. Therefore, it is essential to elucidate which T2Rs are involved in the GLP-1 secreting event of the bitter taste medicinal plants (or their active compound) in the enteroendocrine L cells.

### 3.3. Cannabinoid Receptor

Cannabinoids are psychoactive compounds derived from the body of humans and animals, including endocannabinoid, such as 2-oleoylglycerol (2-OG), or plants, including phytocannabinoids, such as tetrahydrocannabinol (THC). While the cannabinoid receptor type 1 (CB1) and type 2 (CB2) are predominantly expressed in the central nervous system (CNS) and are involved in the hunger signal, GPR119 is predominantly expressed in the pancreas and GI tract and is involved in anorexigenic hormone release such as GLP-1 [59].

GPR119 is known to function as a cannabinoid receptor in human and rodent enteroendocrine K and L cells and pancreatic islets [60–62]. Therefore, GPR119 is expected to play a critical role in glucose homeostasis and incretin secretion. Indeed, a GPR119 null mouse showed reduced GLP-1 secretion in response to oral administration of glucose [63].

The cellular mechanism for GPR119 has not been extensively revealed, but it is believed to couple with G*α*s subunit and to elevate intracellular cAMP levels. GPR119 is assumed to associate with dietary fat-induced satiety because its agonists including 2-OG naturally occur from the digestion of triacylglycerol [40].

On the strength of studies using the human enteroendocrine cell lines and the rodent model, a number of pharmaceutical companies have tried to develop synthetic GPR119 agonists such as PSN-821 (phase 2), MBX-2982 (phase 2), and GSK1292263 (phase 2) for therapeutic purposes (the information of each clinical trial can be accessed through the https://clinicaltrials.gov/ website).

However, the effect of the synthetic GPR119 agonist in human trials is still controversial. AR231453 showed no GLP-1 secreting effect in a human primary enteroendocrine cell culture and JNJ38431055 and GSK1292263 showed limited therapeutic effects on T2DM patients [64, 65], while they stimulated incretin secretion in enteroendocrine cells and a rodent model [63, 66, 67]. These crucial differences between the human clinical trial/human primary cell culture and the rodent model/cell line have presented severe limitations in drug developmental studies.

The agonist for GPR119 is an attractive therapeutic target due to its presence in both the enteroendocrine cells and pancreatic *β* cells. Therefore, the finding of phytocannabinoids (plant derived), which affect only GPR119 but not CB1 or CB2, could be a splendid strategy to treat T2DM. Since the GPR119 agonists are able to regulate both food intake and glucose homeostasis, they have a possibility of serving as efficient therapeutics for metabolic disorders such as obesity and T2DM.

Gordonoside F is a steroid glycoside isolated from African cactiform* Hoodia gordonii*, which has been used for Xhomani Bushmen as an anorexiant during hunting trips [31]. In a recent study using the GPR119 knock-out mice, oral administration of a* H. gordonii* extract (1000 mg/kg) and its active compound Gordonoside F (200 mg/kg) stimulated glucose-dependent GLP-1 and insulin release, showed a blood glucose lowering effect during an oral glucose tolerance test (OGTT), and decreased the cumulative food intake GPR119-dependently [31].

### 3.4. Bile Acid Receptor

Bile acids are steroid acids responsible for biliary lipid secretion, cholesterol elimination, and facilitating the absorption of fat-soluble vitamins and digested dietary lipids into the small intestine [68]. Bile acids are primarily synthesized in the liver and metabolized by gut microflora from the colon. Humans primarily synthesize cholic acid (CA) and chenodeoxycholic acid (CDCA) from the liver, and the CA and CDCA are changed into various secondary bile acids such as deoxycholic acid (DCA), lithocholic acid (LCA), or ursodeoxycholic acid (UDCA).

Bile acids have been reported to bind to the farnesoid X receptor (FXR; also known as bile acid receptor, BAR) and TGR5 (also known as G protein-coupled bile acid receptor 1, GPBAR1). FXR, a nuclear receptor in the liver and intestine, is involved in triglyceride metabolism, glucose metabolism, and liver growth [69].

TGR5, a membrane type G protein-coupled bile acid receptor, is likely involved in metabolic events in the body. Similar to GPR119, TGR5 couples with G*α*s protein and thus elevates the intracellular cAMP levels by binding bile acids [70]. TGR5 expression was found in mouse enteroendocrine STC-1 cells and human enteroendocrine NCI-H716 cells. In the STC-1 cells, LCA and DCA treatment stimulated GLP-1 secretion through TGR5 [71].

In human enteroendocrine NCI-H716 cells, the ginsenoside C-K has been reported to stimulate GLP-1 secretion TGR5 dependently [32]. A notable point about ginsenoside C-K is that it is naturally produced by other ginsenosides, such as Rb1, Rb2, Rc, and Rd, by the metabolic process of the gut microflora [32, 72].

The structural alteration of the phytochemicals by the gut microflora confers the chance to change the binding motif to the expected nutrient sensor. This point provides extended understanding to the investigation of medicinal plant as GLP-1 secretagogues. Perhaps variables such as the gut microflora metabolism that occur* in vivo* cause different results between the cell line/animal studies and human trials.

## 4. Conclusion

As has been reviewed, medicinal plants have an enormous possibility of being developed as GLP-1 secretagogues. They contain abundant bitter tastants, which can act as bitter taste receptor agonists, unexplored phytocannabinoids, which are possible GPR119 agonists, and glycosidic compounds, which are possible sweet taste receptor agonists. Moreover, the structural alteration of the aglycone structure by the gut microflora provides various binding motifs to the exiting and/or deorphanized intestinal nutrient sensors.

The existence of GPR119 in both enteroendocrine cells and also pancreatic *β* cells encourages the development of novel efficient therapeutics for metabolic disorders including obesity and T2DM.

GLP-1 secretagogues stimulate endogenous GLP-1 secretion from enteroendocrine cells. GLP-1 secretagogues do not share the limitations with existing therapeutics for T2DM. Moreover, GLP-1 secretagogues may directly affect obese patients via suppression of food intake.

Considering the tremendous number of phytochemicals and the potential of deorphanized intestinal nutrient sensors, GLP-1 secretagogues from medicinal plants should be actively investigated (Table 2).

## Figures and Tables

**Table 1:** Existing therapeutics for T2DM.

Therapeutics	Limitations	Ref.
*Non-incretin-based therapeutics for T2DM*		
Sulfonylurea	Transiently stimulating excessive insulin secretion that causes hypoglycemia and hyperinsulinemia and eventually results in obesity and overweight.	[16, 17]
Biguanides	Causes gastrointestinal side effects, such as diarrhea and abdominal cramping. Rarely causes lactic acidosis.	[17]
Meglitinides	Transiently stimulating excessive insulin secretion that causes hypoglycemia and results in overweight.	[17]
*α*-glycosidase inhibitor	Causes indigested carbohydrates in gut lumen that may result in flatulence and diarrhea.	[18]
Thiazolidinediones	Causes significant water retention that causes edema and heart failure.	[19]

*Incretin-based therapeutics for T2DM*		
GLP-1 agonists	Inefficient to patients with severe *β* cell dysfunction or insulin resistance; low blood glucose reducing effect compared to the existing therapeutics; cause nausea and vomiting.	[20]
DPP-4 inhibitors	Stimulate unnecessary ductal cells that increase risk of pancreatitis; cause nausea; increase the risk of heart failure.	[21]

T2DM, type 2 diabetes mellitus; DPP-4, dipeptidyl peptidase-4.

**Table 2:** Medicinal plants derived GLP-1 secretagogues via intestinal nutrient sensors.

Origin	Testing agents	Effects	Model	Ref.
Sweet taste receptor				
*Panax ginseng*	Rb1	Decrease food intake, body weight, and body fat mass; decrease fasting blood glucose; decrease blood glucose during the IPGTT; increase plasma insulin	HFD obese rats	[22]
		GLP-1 secretion	Human L cell line	[23]
	Rb2	GLP-1 secretion	Human L cell line	[23]
	Re	Reduces fasting blood glucose	*ob/ob* mice	[24]
	Rg3	Insulin secretion Decrease blood glucose during the OGTT	Rodent *β* cell line ICR mice	[25]

Bitter taste receptor				
*Plasmodium falciparum*	Quinine	GLP-1 secretion	Human L cell line	[26]
*Anemarrhena asphodeloides*	EA fr.	GLP-1 secretion	Human L cell line	[27]
*Bupleurum falcatum*	HX fr.	GLP-1 secretion Decrease blood glucose during the OGTT	Human L cell line *db/db* mice	[28]
*Citrus aurantium*	HX fr.	GLP-1 secretion	Human L cell line	[29]
*Gentiana scabra*	Extract Loganic acid	GLP-1 secretion	Human L cell line	[30]
	Extract	Increase plasma GLP-1 and plasma insulin; decrease blood glucose during the OGTT	*db/db* mice	

Cannabinoid receptor				
Hoodia extract	Extract Gordonoside F	Decreases blood glucose during the OGTT; increases plasma GLP-1 and insulin; decreases cumulative food intake	C57BL/6 mice	[31]
		Glucose stimulated insulin secretion	Isolated rat islets	

Bile acid receptor				
*Panax ginseng*	Compound K	GLP-1 secretion	Human L cell line	[32]

HFD, high-fat diet; IPGTT, intraperitoneal glucose tolerance test; OGTT, oral glucose tolerance test; EA fr., ethyl acetate fraction; HX fr., *n*-hexane fraction.
